# Theoretical and practical approaches to improve the performance of local correlation algorithms for volume data analysis and shape recognition

**DOI:** 10.1107/S2059798321001212

**Published:** 2021-03-30

**Authors:** Valeriy Titarenko, Alan M. Roseman

**Affiliations:** a Division of Molecular and Cellular Function, School of Biological Sciences, Faculty of Biology, Medicine and Health, University of Manchester, Manchester Academic Health Science Centre, Manchester M13 9PT, United Kingdom

**Keywords:** local correlation, discrepancy, molecular-density matching, cryo-EM, map fitting, *DockEM*

## Abstract

In this paper, several approaches to be used to accelerate algorithms for fitting an atomic structure into a given 3D density map determined by cryo-EM are discussed. Rotation and translation of the atomic structure to find similarity scores are used and implemented with discrete Fourier transforms. Several rotations can be combined into groups to accelerate processing. The finite resolution of experimental and simulated maps allows a reduction in the number of rotations and translations needed in order to estimate similarity-score values.

## Abstract 2

Several approaches are presented to improve the performance of local correlation algorithms based on prior information about 3D search and target maps.

## 1. Introduction   

The goal of many cryogenic electron microscopy (cryo-EM) studies is to obtain an atomic model or models corresponding to the data and the molecular complexes imaged. Independent of the techniques used to set up data collection (for example single-particle EM or tilt-series tomography), we obtain sets of projections of an unknown 3D structure onto a digital 2D sensor. After much processing of the recorded images, a reconstruction algorithm provides the user with a 3D density map. In cryo-EM we image with electrons, so the map obtained is strictly an electron potential map. However, it is very similar to an electron-density map that would be obtained by X-ray crystallography, so we will call it a density map by analogy and for convenience. Many software packages have been developed by large teams worldwide to assist with the processing and reconstruction steps, in which all possible information about the experiment is used to determine the best quality density map; see the Collaborative Computational Project for Electron cryo-Microscopy (CPP-EM; Burnley *et al.*, 2017 ▸).

In favourable cases cryo-EM has produced maps to genuine atomic resolution (1.2 Å; Nakane *et al.*, 2020 ▸; Yip *et al.*, 2020 ▸); however, 3–4 Å is more typical of a high-resolution result, and more maps in the lower resolution range between 4 and 8 Å are being published than high-resolution maps.

For higher resolution maps (<3.5 Å) it is possible to derive or build atomic models directly from the map, as individual atoms are resolved or the expected features of protein side chains can be seen and modelled (Emsley & Cowtan, 2004 ▸; Liebschner *et al.*, 2019 ▸). The other case is when the 3D map is at a lower resolution and the features are not rich enough for a structure to be built directly into the map. Of course, there are intermediate situations in which some parts of the map are better than others. In some cases a reliable protein backbone trace can be defined, but most of the side chains are not identifiable.

When an atomic model cannot be built directly into the experimental map manually, if not automatically, it is possible to build a model from larger known fragments (Roseman, 2000 ▸). In a typical case, domains or parts of the molecules might have been solved by X-ray crystallography or nuclear magnetic resonance, or they might be related by homology to parts of other structures that have already been solved. The problem to be solved is the docking of these molecular shapes into the larger map of the entire complex to assemble a model using 3D molecular-density docking. Ideally, objective and quantitative computational methods are needed, with provision for user interaction. At low resolution it may often be necessary to include some additional information to create an unambiguous model, such as constraints from chemical cross-linking or the known effects of mutations.

A complete search of all parameters to locate or dock a particular search density into a larger target map is computationally expensive and time-consuming. It is a large six-dimensional search covering three spatial dimensions and three orientation parameters.

Docking problems in electron microscopy (EM), *i.e.* locating a known density object optimally within a larger 3D density map, are similar to but different from molecular-replacement (MR) problems in X-ray crystallography. The maps produced by cryo-EM techniques are the final results, and no further atomic model-building steps are used to improve the maps. EM images contain phase information and therefore the resultant 3D maps have good-quality phases and amplitudes in Fourier space. However, in some recent programs EM maps are improved by sharpening based on local *B*-factor estimates computed from comparisons with a fitted model.

MR is a method to solve the phase problem in X-ray crystallography, which has a lack of experimental phases in standard diffraction data. In MR, a model or a partial model located in the crystallographic cell is used to obtain some partial phases that can then be used to calculate an initial map. Since the initial map will not have good, if any, phase information, the problem is more difficult than EM molecular docking, and many inventive procedures have been used to solve such problems (see, for example, Colman & Fehlhammer, 1976 ▸). MR is routine if a model similar to the crystallized structure is available and is more challenging when there is not any available model known to be similar to the structure. A correct fit with even a partial fragment can provide sufficient phase information to enable, with further bootstrapping, using more fitting or direct methods, the solution of the correct high-resolution electron-density map to be obtained. For a more complete review of MR, see Evans & McCoy (2008 ▸) and references therein. A similarity of our EM method and MR is the use of correlation and convolution functions to obtain vectors to locate molecular fragments in a density map (Read & Schierbeek, 1988 ▸).

In this paper, we present a series of algorithm improvements for this intensive computational task of docking two densities. Our aim is to generate useful solutions fast enough that docking can be performed interactively by a user on a high-performance desktop workstation. This will allow them to explore and guide model building using additional information and allow them to test their hypotheses in real time.

Our second aim is to facilitate low-resolution model building for another case: where no known candidate homologous structures can be identified for a target map or some region of the map. We propose that with the speedup in computation that we are achieving, a full search of a set of nonredundant molecular fragments from the Protein Data Bank (https://www.wwpdb.org) could be performed. A similar strategy is used for molecular replacement in X-ray crystallography, for example in *MoRDa* (an automatic molecular-replacement pipeline; Vagin & Lebedev, 2015 ▸), which is part of the *CCP*4 online web services (Winn *et al.*, 2011 ▸). Depending on the resolution and character of the structure in the map, the fragments will range in size from domains, or subdomain features, to short peptide-chain fragments of a few amino acids.

The set of candidate domains from the PDB could contain hundreds of thousands of potential structures; however, they may be reduced by removing those with similar features to generate a nonredundant set. Furthermore, we may select those with a given taxonomy, from a given organism, with a given polymer type, with a given enzyme classification *etc*. Some experimental maps have clearly defined domain boundaries, allowing us to split the whole map into smaller blocks, and will give constraints on the fragment size. Therefore, the user could select atomic models to fit based on an appropriate size range. This approach may allow us to limit the number of candidates to thousands. Fitting these models requires the rotation and translation of a large set of search maps in 3D, and is still a computationally intensive task. However, several approaches discussed in this paper allow us to reduce processing times.

In Section 2 we discuss how to define the similarity of two maps. For this purpose, we calculate maps of correlation coefficients and discrepancies. Without any restrictions these similarity scores have a simple relation. However, in the case of noisy data or low signals, extra restrictions can be introduced and the scores are changed. In Section 3 the problem of fitting a smaller 3D map onto a larger map is reviewed. Technical details of this problem with a discussion of methods for optimization are presented in Section 4. Quicker computation of good estimates of correlation maps can be found by reducing the sampling level at the inverse transform stage: see Section 5. Performance numbers for the *DockEM* program modified with the use of the proposed ideas and comparison with the *PowerFit* program are presented in Section 6.

## 2. Similarity of vectors for noisy data   

Suppose we have two 1D vectors of size *n*: *f*_*i*_, *g*_*i*_, *i* = 0, …, *n* − 1. The similarity of these vectors can be found in several ways. We use notions of cross-correlation and discrepancy (see Appendix *A* [App appa]). For a normalized signal *g*, *i.e.* 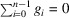 and 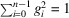, and without any restrictions on the level of signal *g*, the normalized correlation and discrepancy coefficients are defined as 
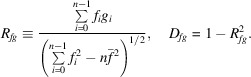



Real data contain noise and errors; therefore, instead of the exact signal *f*_*i*_ we know only some approximations 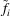 such that 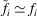. In some cases we know the error values, *i.e.* ɛ_*i*_ > 0 such that 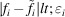. In other cases, we may assume that 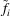 has some distribution with given parameters.

Similarity scores are independent of the amplitude of a signal but are sensitive to the signal-to-noise ratio. In the case of strong signal values of *f*_*i*_ small errors ɛ_*i*_ should not cause any issues when similarity scores are calculated, since ɛ_*i*_/|*f*_*i*_| are small. This means that the scores found for erroneous data 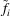 have similar values compared with (unknown) exact values *f*_*i*_. In the case of low signal, for example *f*_*i*_ ≃ 0, ɛ_*i*_/|*f*_*i*_| becomes large, so more solutions with the given error are possible and a higher variability of similarity scores is observed. As a result, we may find a lot of false matching of two signals. Therefore, it is reasonable to put some additional restrictions on signal *f*_*i*_.

Suppose a signal is given on a subvolume. To avoid false peaks for similarity values, we require the signal to be different from noise. However, as similarity scores do not change if we vary the mean values, we may obtain false peaks even when a signal is close to a nonzero constant. Thus, we need to force the signal to be different from its mean value for the given subvolume. Therefore, we may threshold the variance, *i.e.* set a number σ_0_ > 0 and redefine the correlation coefficient as 
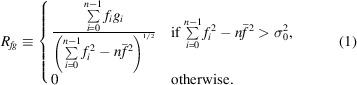
This means that for low-variance regions of the target map we set the correlation coefficient to zero.

For the discrepancy we may require the scaling factor α to be greater than a given number α_0_. In this case, the minimum of (6) is attained at the point α = α_0_, 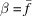 if 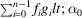. Therefore, 
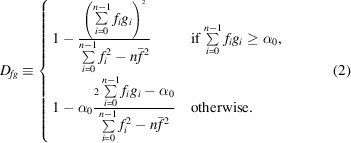
The times taken to calculate *D* and *R* similarity scores are almost the same.

## 3. Correlation map in 3D   

Suppose we have a target map *T* (a density distribution after cryo-EM pre-processing and reconstruction). There is also a smaller search map *S* (this map is generated from known atomic structures). Our goal is to position the search map *S* onto the target map *T* in such a way that their common parts are similar. We also introduce a mask to confine the signal compared to a fixed region around the search object, reducing the noise contributed by unmatched parts of the target map (see Roseman, 2003 ▸). Let us consider a 1D case first.

There are vectors *T*_*i*_, *i* = 0, …, *N* − 1 and *S*_*j*_, *j* = 0, …, *K* − 1. In general *N* and *K* are different. We choose an index *q* and consider a subvector *T* ^*q*^ of length *K* of vector *T*: *T*_*q*+*j*_, *j* = 0, …, *K* − 1. The subvector *T* ^*q*^ and vector *S* are of the same length *K*. We may find similarity scores for those two vectors as described in the previous section. For example, the corresponding correlation coefficient is 
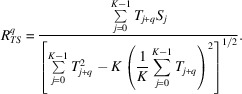
The length of vector *S* is *K* and the length of vector *T* is *N*; therefore, there are only *P* = *N* − *K* + 1 common segments when all elements of vector *S* and subvector *T* ^*q*^ are known.

In principle, the search signal may be defined on multiple segments. Therefore, it is better to extend the search vector and define it for all integer indices. At the same time, we may introduce the mask vector *M*_*j*_ such that *M*_*j*_ = 1 when *S*_*j*_ is known and *M*_*j*_ = 0 otherwise. Note that *S*_*j*_ = *S*_*j*_ *M*_*j*_. We define the number of known elements as *w*, *i.e.* 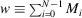. The correlation coefficient can be written as 
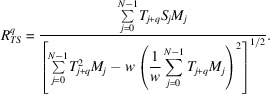



If there are two discrete (real-valued) functions *f*_*i*_, *g*_*i*_, *i* = −∞, …, ∞ then their cross-correlation is defined as 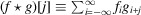. We may set the *T*, *S*, *M* vectors to zero outside *i* = 0, …, *N* − 1; the correlation coefficient at point *q* can then be written as 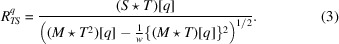
In a similar way, we may find 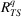 and 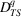 in the case of erroneous data by using (1) and (2). For instance, if 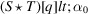, then 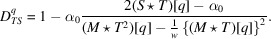
These expressions for similarity values written with the use of cross-correlation functions are left unchanged if we consider 3D maps.

The main difference from the fast local correlation algorithm introduced for 2D maps (images) in Roseman (2000 ▸) is the use of cross-correlation operations instead of convolutions. While the results are the same in the case of symmetric maps, they are different for arbitrary-shaped search maps.

Let the target vector *T* be of length *N* and the search vector *S* be of length *K*; we then pad them with zeros and make them periodic with period *L*. If we use (7) to find the cross-correlation vector 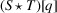 then it will have the correct values only for *q* = 0, …, *L* − *K*.

Suppose we are given 3D maps and want to find similarity scores over some region of interest (ROI). We should expand the ROI by the size of the search map. We can then use a 3D DFT (discrete Fourier transform; see Appendix *A*) for the target/search maps on the extended ROI and find cross-correlation with (7).

## 4. Numerical implementation   

Here, we discuss technical details that help us to improve the performance of the algorithm defined by (3). A key feature of the procedure is that the correlation coefficient is computed in a possibly irregular, local area defined by a mask enclosing the search object.

### 4.1. Size of maps   

Suppose we are given a target map *T* of size 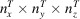. We also know an atomic model that we aim to fit at some points of the target map. The atomic model can be given as the coordinates of atoms in a PDB file. We then simulate the density distribution corresponding to the given atomic model, so that we have two equivalent-sized maps, which will allow the correlation calculation to be performed using FFTs.

An electron potential map is generated using a five-Gaussian approximation for each atom (see Appendix *A* [App appa]). In this way, we can generate an *S* map for an arbitrary small voxel size. For now, we assume that the sampling of the *T* and *S* maps are the same. Suppose that there is a procedure to generate the mask map *M* from our search map *S*.

For given *T*, *S* and *M* maps we apply an algorithm to find the score values for each point of map *T*. However, by default we do not known the orientation of the atomic structure. Therefore, the algorithm to find score values should sample all orientations. Of course, the final results should not depend on the position of the centre of rotation of the atomic structure. In principle, we may choose any point as a centre of rotation, as long as the ROI is large enough to accommodate the rotations. A convenient and intuitive way is to use the centre of mass of the molecule. However, to keep the rotated maps in a smaller volume, it is more efficient to use a point close to the centre of the bounding sphere; see Gärtner (1999 ▸) and Larsson (2008 ▸). In this way, the size and the shape of the corresponding *S* and *M* maps can be kept the same independent of an initial chosen orientation of the atomic structure.

In principle, the centre of rotation can be anywhere (not always at a corner of a voxel for *S* and *M* maps). However, keeping the centre at an integer point (an integer number of pixels for each direction) may help us to implement some extra procedures to improve performance.

Let maps *S*, *M* have size 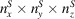 (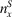, 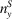, 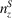 are even numbers) and the centre of rotation be at 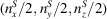. Suppose the maps are padded with zeros in such a way that no nonzero values will be outside these maps if they are arbitrarily rotated. We may want to find score values within the box [*x*_*s*_, *x*_*e*_] × [*y*_*s*_, *y*_*e*_] × [*z*_*s*_, *z*_*e*_], where *x*_*s*_, *x*_*e*_, *y*_*s*_, *y*_*e*_, *z*_*s*_, *z*_*e*_ are integer numbers. To find the score values for this ROI, we need to use the following ROI for *T*: 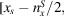 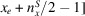 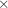 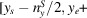 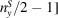 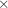 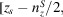 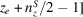, which should be inside 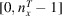 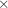 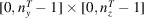. As the size of Fourier images for *T*, *S* and *M* should be the same, we need to pad them with zeros. The least size of the padded maps is 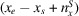 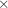 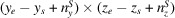.

There are many libraries providing users with DFT implementation. The general rule for the time required to perform a 1D DFT is 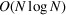, where *N* is the size of the vector. However, each implementation may provide different numbers depending on the computational architecture (CPU/GPU, memory bandwidth). In Gambron & Thorne (2020 ▸) a performance comparison of most popular FFT libraries is made. The most critical factor is the domain size. We see that execution times vary significantly depending on *N*. When the prime decomposition of *N* contains prime numbers larger than 5 the time taken is disproportionately longer, but is otherwise approximately linear with 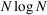. Therefore, in some cases it is better to increase the domain (with zero padding) to achieve better run times. In any case, keeping the centre of rotation for *S*, *M* maps as close to the centre of the bounding sphere helps us to reduce the whole size of Fourier maps for *T*, *S* and *M*.

### 4.2. Rotation and Fourier space   

To compute the correlation map we need to perform three cross-correlations: 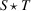, 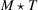 and 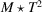. Therefore, we need to perform four forward DFTs, for *M*, *S*, *T* and *T* ^2^, and three backward DFTs, for 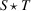, 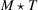 and 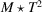. As maps *T* and *T* ^2^ are fixed, 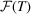 and 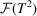 can be performed only once (before any rotations). Thus, for each rotation we need to perform five DFTs.

The uncertainty principle is valid for the Fourier transform. The smaller the support of a function *g* is (*i.e.* where it has nonzero values), the more spread out its Fourier transform 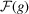. The support of *S* and *M* is relatively small with respect to the extended ROI of *T*. Thus, we should expect the corresponding Fourier images of *S* and *M* to be dense maps. While finding a 3D DFT of a rotated object can be performed with lower dimension DFTs of the original (nonrotated) Fourier map (Paeth, 1990 ▸; Larkin *et al.*, 1997 ▸), this is a quite time-consuming operation which at the same time introduces extra errors due to interpolation procedures. Therefore, it is faster to find *S* and *M* maps with finer sampling and then perform remapping with nearest-neighbour or linear interpolation for given Euler angles onto a map of the same voxel size as the *T* map.

### 4.3. Cubic ROIs and an integer centre of rotation   

When we try to fit an atomic model (or some density search object) to an area of a target map, the general rule is to rotate the *S* and *M* maps while keeping the *T* map fixed. Search and mask maps can be found with high accuracy in a relatively short time; therefore, we may assume that there are almost no interpolation errors for rotated *S* and *M* maps.

Rotating target maps usually introduces interpolation errors; therefore, the maps of correlation or discrepancy scores may also be susceptible to these errors. However, we may avoid any interpolation errors if the centre of rotation is at an integer point (*i.e.* the centre of rotation of *S*, *M* is at a corner of one of the voxels of the *T* map). If we rotate by 90° around a coordinate axis, then the indices of the new map are integer numbers, *i.e.* are also indices of the original map. Therefore, we may find all possible perfect rotations of *T* and use multiple *T* maps for the same rotated *S*, *M* maps. If the ROIs are cubic, then all rotated maps have the same shape.

**Figure 1:**
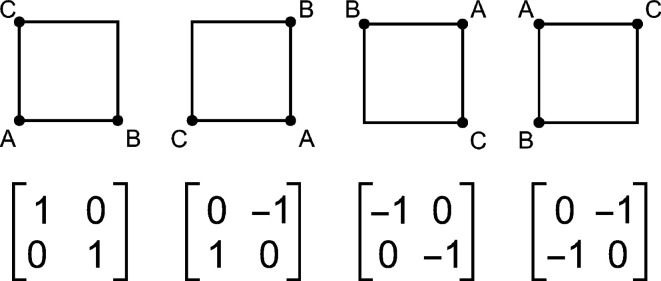
2D rotations of a square and the corresponding rotation matrices.

In the 2D case there are four such rotations for a square. The rotations and corresponding matrices are shown in Fig. 1 ▸. If the original map has a pixel with indices (*i*, *j*), then with the rotation matrix 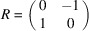
the new index is (*i*_new_, *j*_new_) = (−*j*, *i*). All indices should be from 0 to *n* − 1. Due to the periodicity of all maps (required to perform circular discrete correlation with DFT), then *i*_new_ = *n* − *j* if *j* > 0 and *i*_new_ = 0 if *j* = 0; *j*_new_ = *i* for any *i*.

**Figure 2:**
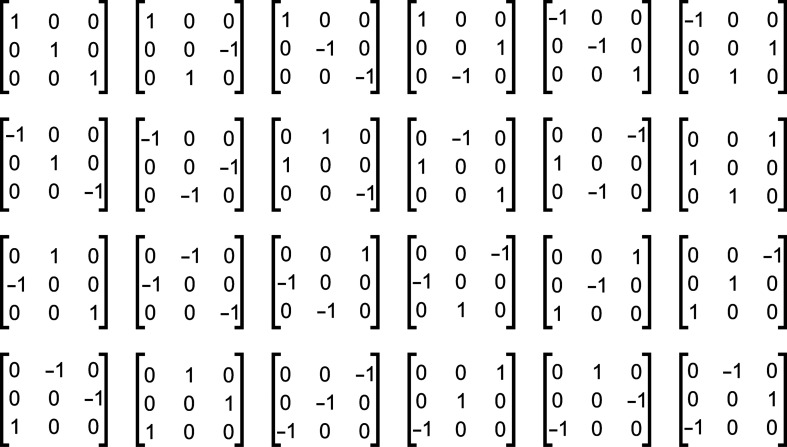
Matrices for 3D orthogonal rotations to relate the different octants to each other.

In 3D we may use properties of *P*432 symmetry for a cube. There are 24 symmetry operations (see Aroyo, 2016 ▸). The corresponding rotation matrices are shown in Fig. 2 ▸.

Suppose for a given set of Euler angles defining rotation matrices we may combine them into a group of 24 matrices (each matrix is a matrix product of one matrix with one of the matrices defined in Fig. 2 ▸). If we do not combine these rotation matrices into groups of 24, then to find a correlation map we need to perform 5 × 24 = 120 DFTs for each 24 rotations. However, if these matrices are combined then we just need to perform two forward DFTs for *S* and *M* and 3 × 24 = 72 backward DFTs for 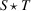, 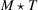 and 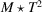, *i.e.* 74 DFTs in total or 61.7% of 120.

## 5. Reduction of the output map   

### 5.1. False peaks   

If we have errors in the input data, then there is a chance that a correlation coefficient 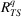 may have peaks in areas where values of *T* are small, since both the numerator and denominator in (3) are small numbers that are sensitive to small errors in the *T*, *S* and *M* maps. Therefore, in practical applications we first estimate the maximum value of 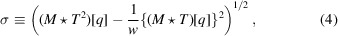
for all possible rotations and translations of *S*, *M* maps. We then set σ_0_ in (1) as a percentage of the maximum value of σ.

**Figure 3:**
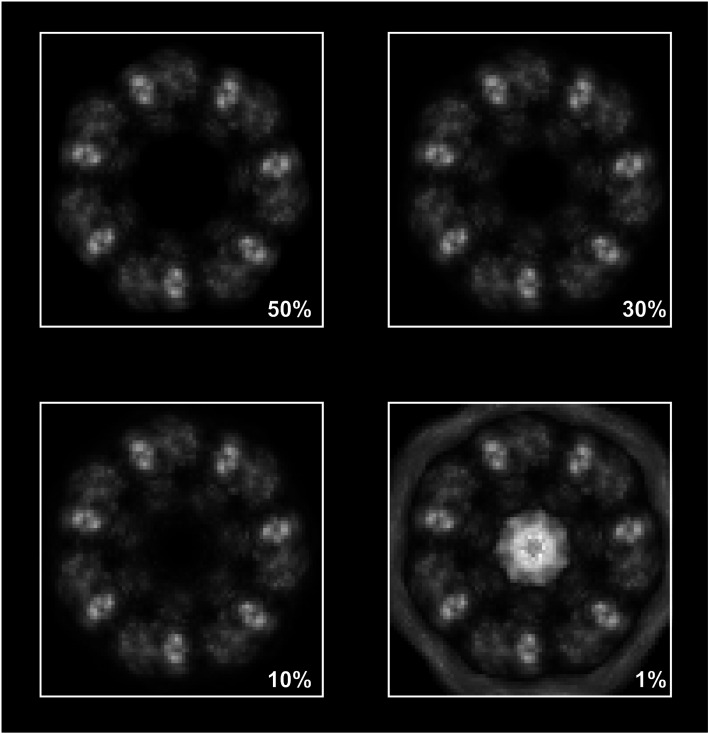
Correlation maps obtained for different levels of standard deviation of the signal (50%, 30%, 10%, 1%).

We demonstrate our procedure by fitting an atomic structure (the intermediate domain, which at ∼9 kDa is the smallest of three defined domains in the GroEL subunit) into an EM map of the GroEL chaperonin complex (EMDB entry EMD-1997, resolution 7 Å, *C*7 symmetry; Clare *et al.*, 2012 ▸). The domain was extracted from PDB entry 2c7e (residues 136–191 and 374–409; Ranson *et al.*, 2001 ▸). We find the maximum value of σ and then set σ_0_ as 50%, 30%, 10% and 1% of σ. For a slice perpendicular to the axis of symmetry we calculate 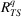 (see Fig. 3 ▸). One may see that for σ_0_ ≥ 0.1σ_max_ the local maximum values of the correlation maps are the same. On decreasing σ_0_ more peaks start to appear in the central and outer parts of the map where the target map *T* is almost zero. When σ_0_ is about 1% of σ_max_ the central part of the map has higher correlation coefficient values than the real peaks. By setting σ_0_ to a correct level we reduce extra checking of these false peaks as centres of the chosen atomic structure.

A similar idea can be used in the case of a discrepancy map when we need to set the minimum level of α and apply formula (2).

### 5.2. Smooth functions   

Maps *T* and *S* are both smooth functions, *i.e.* their neighbouring voxels should have similar values. Therefore, the 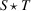 map is also a smooth function. In many practical cases *M* is a set of a small number of convex or almost convex shapes, so we may also expect 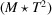 and 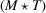 to be smooth functions.

To avoid false peaks in the 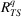 map we set σ ≥ σ_0_. As a result, 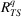 is also a smooth function of *q* for these areas. Thus, to identify areas where 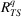 has peaks we may not need to find 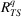 at each voxel, especially when we use a brute-force approach and have to check many rotation angles. For instance, we may find the map only at even voxels when all three indices of a voxel are even numbers. In this case the size of the output map is reduced eight times.

Of course, we may try to reduce the number of voxels for all maps (*T*, *S* and *M*). However, as some original voxels are skipped this led to maps that differed from the original ones. Our goal is to have the same accuracy of the output maps but just find values at a reduced number of voxels.

### 5.3. Reduced maps   

Suppose we have an *N*-vector *G*_*k*_ in Fourier space and *N* = *mK*, *m* and *K* are integer numbers. We want to find the values of the corresponding vector *g* but only at indices *q* = *mn*, *n* = 0,…, *K* − 1. By the definition of backward DFT we find 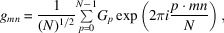


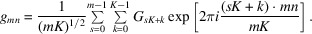
Taking into account that exp(2π*isn*) = 1 we obtain
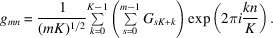
If we denote 
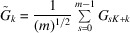
then 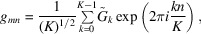

*i.e.* each *m*th element of vector *g* is the backward DFT of vector 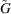.

Similar formulae are valid for 2D and 3D DFTs. We just need to add up the values for corresponding slices of a 3D map, thus reducing the size *m* times. The procedure is then continued for rows and columns, so the final volume is reduced *m* ^3^ times. This procedure is straightforward in the case of complex input/output maps.

### 5.4. Real and complex DFTs   

We usually have real-valued *T*, *S* and *M* maps, so in many software libraries more compact data storage can be used to store the corresponding complex maps 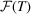, 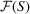 and 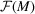 in Fourier space. If *g*_*n*_, *n* = 0, …, *N* − 1 is a real-valued vector, then for the corresponding *G*_*k*_ vector in Fourier space we obtain 
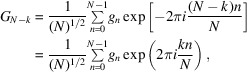


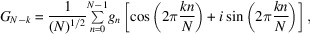


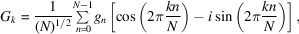
so 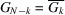, 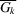 is the complex conjugate of 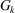. Therefore, we may need to store almost half of vector *G*. This leads to more processing if we want to reduce the output maps.

However, we may work with complex maps (both for input and output maps). There are several ways to combine real maps into complex maps.

(i) *S* and *M* form a complex map (*S*, *M*). Instead of real maps *T* and *T* ^2^, we create complex maps (*T*, 0), (*T* ^2^, 0). Then, 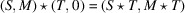, 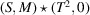 = 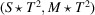. This approach requires us to double the storage size to store 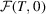, 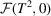 and we obtain an additional output map 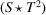 which is not used to find score values.
(ii) We use (*T*, *T* ^2^) and (*S*, 0), (*M*, 0). Therefore, 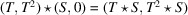, 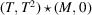 = 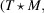 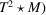.
(iii) If several maps *T* are given, *i.e.* 12 pairs of 24 pre-rotated *T*, then (*T*_1_, *T*_2_), 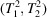 are cross-correlated with (*S*, 0) and (*M*, 0).

Finding 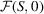 and 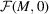 with the use of DFT for a complex map takes twice the time needed for the real-valued version of DFT for 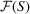 and 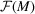. However, we may just use 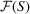 and 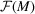 maps created in a compact form and double their sizes with the horizontal flipping of corresponding rows, therefore (depending on the given CPU/GPU architecture and the libraries available) this operation should not take a lot of time. The processing time for backward DFTs should be roughly proportional to the number of backward DFTs multiplied by the factor 1/*m* ^3^.

DFT algorithms are used to find convolution or cross-correlation maps in a faster way when the corresponding elements of maps in Fourier space are processed in some way, for example multiplied. There is usually no need to access neighbouring elements in Fourier space. As a result, storage schemes for data in Fourier space may be organized in such a way as to achieve the highest throughput. This may lead to the neighbouring elements in memory not being the neighbouring elements for the data in Fourier space. Therefore, the data-reduction process in Fourier space may not be straightforward as is described above and may depend on a particular implementation of a DFT algorithm.

Assuming (i) that finding a DFT for a complex map (*A*, *B*) takes twice the time and space compared with a DFT for a real-valued map *A* or *B*, (ii) that there is no time wasted in reducing the output map in Fourier space and (iii) that the time to process the reduced map is 1/*m* ^3^ of the original time, then we obtain theoretical times and storage requirements to process *N*_*G*_ (*N*_*G*_ is an even number) maps *T* (see Table 1 ▸). Therefore, we can estimate the time required to find the correlation maps for *N*_*G*_ rotations: 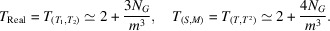
For example, for a *DockEM* implementation (Windows machine, six-core Intel Core i7-3930K CPU, Intel’s performance IPP and MKL libraries) the time to process *N*_*G*_ = 24 rotations and provide a reduced output map (*m* = 2) should be approximately 2 + [(3 × 24)/2^3^] = 11 compared with the *m* = 1 case of 74, *i.e.* 15%. However, in practice it was only possible to achieve 50% due to the extra time needed to process the Fourier images of original maps in order to obtain Fourier images of the reduced maps. Nevertheless, the combined performance improvement when 24 rotations are combined into one group was about three times compared with a general case of single rotation with a full-size output map.

## 6. Run times   

The *DockEM* code considers all possible 3D rotations without any restrictions. There are several approaches (to be discussed in other papers) to generate an optimal number of rotations for a given angular resolution. Suppose that there is a set of rotations, and for any other possible rotation we try to find the nearest rotation from the given set. The maximum possible angular distance for any possible rotation is the angular resolution of the given set of rotations. As the best performance is achieved for rotations that are combined into groups, for each near rotation we also have other 23 rotations. Table 2 ▸ shows the number of rotation groups for a given angular resolution.

To illustrate a couple of use cases, we used the EMD-1997 map of GroEL (7 Å resolution, *C*7 symmetry, sampled at 2.02 Å per pixel) and the atomic structure from PDB entry 2c7e mentioned before (the intermediate domain, which is the smallest and the most difficult to fit), and ran the code on a ten-core processor (Intel Core i9-7900X, 3.30 GHz). Searches were run at 7 Å resolution. In the first example we used a target map region size of 70 pixels, corresponding to a full search of the whole molecule. The size of the mask defining the search object was 28^3^. The angular resolution was set to 3°, *i.e.* we need 19 382 groups of 24 rotations. It took 1707 s (28.5 min). We can estimate this time using Table 3 ▸. For a 24^3^ mask and a 64^3^ target we obtain 1.44 min for 1000 groups, so 19.382 × 1.44 = 27.9 min.

*DockEM* is one of many algorithms for atomic fitting. A list of most the popular methods can be found in Villa & Lasker (2014 ▸). A full comparison with other methods will be performed in future papers, as many factors related to performance, quality of fitting, resolution range and choice of orientations need to be taken into account. *PowerFit* (van Zundert & Bonvin, 2015 ▸) is one of several programs based on similar ideas to *DockEM*. Here, we provide performance numbers to compare these programs. In van Zundert & Bonvin (2015 ▸) for a 128^3^ problem (the combined size of the target and search maps) a fine (4.71°) rotational search was performed on an Intel Core i7-3632QM CPU (four cores, 2.20 GHz frequency). The authors used 70 728 pre-calculated orientations. It took them 6 h 23 min or 383/70.728 = 5.41 min for 1000 orientations. According to Table 3 ▸, for a 128^3^ problem (*e.g.* a 96^3^ target map and 32^3^ search map) we obtain 3.84 min for 1000 groups of 24 rotations (or 0.16 min per 1000 rotations) on an Intel Core i9-7900X (ten cores, 3.30 GHz). As different CPUs were used, we assume that *PowerFit* run times are inversely proportional to the number of cores and the CPU frequency, so on an Intel Core i9-7900X CPU we would expect *PowerFit* to process 1000 rotations in 5.41 × 2.2 × 4/(3.3 × 10) = 1.44 min. This means that *DockEM* performs 1.44/0.16 = 9 times faster than *PowerFit*.

The current version of the code can be used for interactive fitting. Suppose that we have roughly positioned the rotation centre of the search object within a 16-pixel cube, and the size of the search object can also be placed in a 16-pixel cube. We then use a 32^3^ region of the target map to obtain correct score values with the 16^3^ volume. If the angular resolution is 4°, then according to Table 2 ▸ we need to use 7568 groups of rotations. It took us 9.3 s. For a coarser angular fitting the processing time is within the range 3–4 s.

## 7. Conclusions   

Fitting an atomic structure into a medium- or low-resolution map may require several processing steps.

The approach used depends on the research question and the data available. For example, there may be models or known atomic structures of fragments or domains for your structure in the PDB, or you may have a homology model. The problem is essentially then a 3D puzzle to assemble the known fragments into the 3D density map.

In another case, there may be no identifiable sequence homology with any solved structure. A search of selected candidates from the PDB could then be performed. Filters on criteria such as mass or secondary-structure composition could be applied to reduce the number of domain structures to search with.

EM density maps have some finite resolution which allows us to estimate the smoothness of maps for similarity scores. To avoid false peaks of similarity values we may put some restrictions on the level of signal. This helps to save time in checking some positions and orientations of the atomic structure and ill-defined, or solvent, regions of the target map. Smoothness of the given and simulated maps allows us to skip some neighbouring orientations as well as to avoid finding score values at all points of the original map.

Positioning the centre of rotation of a given atomic structure at specific points enables us to combine several (up to 24) rotations in one group, which increases performance. Combining several real maps into complex maps may also be an efficient approach but depends on particular data-storage schemes and software libraries, which will dictate how efficiently a map-reduction algorithm can be implemented.

We have shown that we can obtain significant improvement in the efficiency and speed of the correlation searches without losing accuracy. These can be used to implement real-time interactive programs or tools that will be useful for researchers. In addition, we are optimistic that we can apply these techniques to interpret lower resolution cryo-EM maps (which are not suitable for other model-building methods) by identifying and positioning matching fragments from the PDB. 

## Figures and Tables

**Table 1:** Storage requirements and number of DFTs to find correlation maps for *N*_*G*_ rotations One single single storage unit is the size of {\cal F}(T); one DFT is the time to calculate {\cal F}(T).

Option	Storage for {\cal F}(T), {\cal F}(T^{2}); {\cal F}(T,0), {\cal F}(T^{2},0)	Storage for {\cal F}(S), {\cal F}(M); {\cal F}(S,0), {\cal F}(M,0)	No. of forward DFTs	No. of backward DFTs
Real	2*N*_*G*_	2	2	3*N*_*G*_
(*S*, *M*)	4*N*_*G*_	2	2	4*N*_*G*_
(*T*, *T* ^2^)	2*N*_*G*_	4	4	4*N*_*G*_
(*T*_1_, *T*_2_)	2*N*_*G*_	4	4	3*N*_*G*_

**Table 2:** Angular resolution as a function of number of rotation groups

Angle (°)	16	12	10	9	8	6	4	3	2
Groups	109	257	439	598	871	2125	7568	19382	87718

**Table 3:** Time (in minutes) to process 1000 groups of rotations Columns, target size; rows, source size.

	32	64	96	128	160	192	224	256
16	0.13	0.89	3.14	6.04	9.96	20.43	18.49	18.60
24	0.22	1.44	3.84	6.50	13.32	19.56	23.36	23.25
32	0.47	1.58	3.69	8.62	12.41	22.82	30.37	32.27
48	0.90	3.14	6.12	9.88	20.53	33.44	43.58	48.66

## Appendix A

### A1. Correlation coefficient and discrepancy

Let there be two vectors of size *n*: *f*_*i*_, *g*_*i*_, *i* = 0, …, *n* − 1. The correlation coefficient is defined as

where  and  are the mean values for the vectors, *i.e.* . For a given arbitrary vector , we may define vector *g*_*i*_ such that

In this case  and , and the original formula (5) can be rewritten as

The correlation coefficient has a range [−1, 1]. If *f*_*i*_ = *g*_*i*_ then *R*_*fg*_ = 1, and in the case of *f*_*i*_ = −*g*_*i*_, *R*_*fg*_ = −1.

We allow a signal to be scaled and translated by constant values. For constants α and β a discrepancy is defined as

We try to vary the parameters α, β so the discrepancy attains its minimal value. The smaller the value of the discrepancy is, the more similar the vectors *f* and *g* are.

If, as above, we assume , , then

is a quadratic function of α and β. To find its minimum, we need to solve , . The optimal values are  and  and the minimum value of  is

The discrepancy value is a non-negative number. It is better to normalize it so the range is [0, 1], as

If no restrictions are put on the values of α and β, then the correlation coefficient and the normalized discrepancy have a simple relation:

### A2. Discrete Fourier transform

Let *g*_*n*_, *n* = 0, …, *N* − 1 be complex numbers. The discrete Fourier transform (DFT) provides us with the vector *G*_*k*_, *k* = 0, …, *N* − 1 according to the formula

where *i* = (−1)^1/2^. For brevity, this forward DFT can be written as . The inverse DFT can be written as .

In the case of periodic signals, *i.e.* when *g*_*n*+*N*_ = *g*_*n*_ for any *n*, we may use the important property of the DFT

where  is the complex conjugate of *f*.

### A3. Atomic scattering factors

According to Bai (2003 ▸), for a spherical radial density model the density distribution can be written as

where *f*(*q*) is an atomic scattering factor. In the range of scattering vectors *q* < 25 Å^−1^ the atomic scattering factors can be approximated by a sum of Gaussian functions,

where the coefficients *a*_*i*_, *b*_*i*_, *i* = 1, …, 4 and *c* can be found in Ibers & Hamilton (1974 ▸). Using the above formula and with either tabulated *B* factors from a PDB file or a known resolution for the density distribution, the process of finding the density distribution for the whole molecule is fast and straightforward.

## References

[bb1] Aroyo, M. I. (2016). Editor. *International Tables for Crystallography*, Vol. A, 2nd online ed., pp. 612–613. Chester: International Union of Crystallography.

[bb2] Bai, J. (2003). *Phys. Rev. B*, **68**, 144109.

[bb3] Burnley, T., Palmer, C. M. & Winn, M. (2017). *Acta Cryst.* D**73**, 469–477. 10.1107/S2059798317007859 PMC5458488 28580908

[bb4] Clare, D. K., Vasishtan, D., Stagg, S., Quispe, J., Farr, G. W., Topf, M., Horwich, A. L. & Saibil, H. R. (2012). *Cell*, **149**, 113–123. 10.1016/j.cell.2012.02.047 PMC3326522 22445172

[bb5] Colman, P. M. & Fehlhammer, H. (1976). *J. Mol. Biol.* **100**, 278–282. 10.1016/s0022-2836(76)80063-0 1255714

[bb6] Emsley, P. & Cowtan, K. (2004). *Acta Cryst.* D**60**, 2126–2132. 10.1107/S0907444904019158 15572765

[bb7] Evans, P. & McCoy, A. (2008). *Acta Cryst.* D**64**, 1–10. 10.1107/S0907444907051554 PMC2394790 18094461

[bb8] Gambron, P. & Thorne, S. (2020). *Comparison of Several FFT Libraries in C/C++.* RAL Technical Report RAL-TR-2020-003. Didcot: STFC Rutherford Appleton Laboratory.

[bb9] Gärtner, B. (1999). *Algorithms – ESA’ 99*, edited by J. Nešetřil, pp. 325–338. Berlin, Heidelberg: Springer.

[bb10] Ibers, J. A. & Hamilton, W. C. (1974). Editors. *International Tables for X-ray Crystallography*, Vol. IV, pp. 99–101. Birmingham: The Kynoch Press.

[bb11] Larkin, K. G., Oldfield, M. A. & Klemm, H. (1997). *Opt. Commun.* **139**, 99–106.

[bb12] Larsson, T. (2008). *SIGRAD 2008. The Annual SIGRAD Conference Special Theme: Interaction*, pp. 27–30. Stockholm: Linköping University Electronic Press.

[bb13] Liebschner, D., Afonine, P. V., Baker, M. L., Bunkóczi, G., Chen, V. B., Croll, T. I., Hintze, B., Hung, L.-W., Jain, S., McCoy, A. J., Moriarty, N. W., Oeffner, R. D., Poon, B. K., Prisant, M. G., Read, R. J., Richardson, J. S., Richardson, D. C., Sammito, M. D., Sobolev, O. V., Stockwell, D. H., Terwilliger, T. C., Urzhumtsev, A. G., Videau, L. L., Williams, C. J. & Adams, P. D. (2019). *Acta Cryst.* D**75**, 861–877. 10.1107/S2059798319011471 PMC6778852 31588918

[bb14] Nakane, T., Kotecha, A., Sente, A., McMullan, G., Masiulis, S., Brown, P. M., Grigoras, I. T., Malinauskaite, L., Malinauskas, T., Miehling, J., Uchański, T., Yu, L., Karia, D., Pechnikova, E. V., de Jong, E., Keizer, J., Bischoff, M., McCormack, J., Tiemeijer, P., Hardwick, S. W., Chirgadze, D. Y., Murshudov, G., Aricescu, A. R. & Scheres, S. H. W. (2020). *Nature*, **587**, 152–156. 10.1038/s41586-020-2829-0 PMC7611073 33087931

[bb15] Paeth, A. W. (1990). *Graphics Gems*, edited by A. S. Glassner, pp. 179–195. San Diego: Morgan Kaufmann.

[bb16] Ranson, N. A., Farr, G. W., Roseman, A. M., Gowen, B., Fenton, W. A., Horwich, A. L. & Saibil, H. R. (2001). *Cell*, **107**, 869–879. 10.1016/s0092-8674(01)00617-1 11779463

[bb17] Read, R. J. & Schierbeek, A. J. (1988). *J. Appl. Cryst.* **21**, 490–495.

[bb18] Roseman, A. M. (2000). *Acta Cryst.* D**56**, 1332–1340. 10.1107/s0907444900010908 10998630

[bb19] Roseman, A. M. (2003). *Ultramicroscopy*, **94**, 225–236. 10.1016/s0304-3991(02)00333-9 12524193

[bb20] Vagin, A. & Lebedev, A. (2015). *Acta Cryst.* A**71**, s19.

[bb21] Villa, E. & Lasker, K. (2014). *Curr. Opin. Struct. Biol.* **25**, 118–125. 10.1016/j.sbi.2014.04.001 24814094

[bb22] Winn, M. D., Ballard, C. C., Cowtan, K. D., Dodson, E. J., Emsley, P., Evans, P. R., Keegan, R. M., Krissinel, E. B., Leslie, A. G. W., McCoy, A., McNicholas, S. J., Murshudov, G. N., Pannu, N. S., Potterton, E. A., Powell, H. R., Read, R. J., Vagin, A. & Wilson, K. S. (2011). *Acta Cryst.* D**67**, 235–242. 10.1107/S0907444910045749 PMC3069738 21460441

[bb23] Yip, K. M., Fischer, N., Paknia, E., Chari, A. & Stark, H. (2020). *Nature*, **587**, 157–161. 10.1038/s41586-020-2833-4 33087927

[bb24] Zundert, G. C. P. van & Bonvin, A. M. J. J. (2015). *AIMS Biophys.* **2**, 73–87.

